# Aquaporin-4 expression in the cerebrospinal fluid in congenital human hydrocephalus

**DOI:** 10.1186/2045-8118-10-18

**Published:** 2013-05-09

**Authors:** Leandro Castañeyra-Ruiz, Ibrahim González-Marrero, Juan M González-Toledo, Agustin Castañeyra-Ruiz, Héctor de Paz-Carmona, Agustín Castañeyra-Perdomo, Emilia M Carmona-Calero

**Affiliations:** 1 Departamento de Anatomía, Facultad de Medicina, Universidad de La Laguna, La Laguna, Tenerife, Canary Island, Spain; 2 Departamento de Biotecnología, Instituto de Investigación y Ciencias de Puerto del Rosario, Fuerteventura, Canary Island, Spain; 3 Departamento de Farmacología, Facultad de Medicina, Universidad de La Laguna, Tenerife, Canary Island, Spain

**Keywords:** Aquaporin-4, Congenital hydrocephalus, Cerebrospinal fluid

## Abstract

**Background:**

Aquaporin-4 (AQP4) is a water channel mainly located in the ventricular ependymal cells (brain-CSF barrier), the sub-ependymal glia, glia limitans and in end-feet of astrocytes in at the blood–brain barrier (BBB).

**Methods:**

In the present work, the expression of AQP4 in the cerebrospinal fluid (CSF) in control and congenital human hydrocephalus infants (obstructive and communicating), was analysed by Western-blot and enzyme immunoassay (ELISA).

**Results:**

AQP4 was found to be high compared to the control in the CSF in congenital hydrocephalus patients. Western-blot showed higher values for AQP4 than controls in communicating hydrocephalus (communicating: 38.3%, control: 6.9% *p* < 0.05) although the increase was not significant in obstructive hydrocephalus (obstructive: 14.7%). The AQP4 quantification by ELISA also showed that, the mean concentration of AQP4 in CSF was significantly higher in communicating hydrocephalus (communicating: 11.32 ± 0.69 ng/ml, control: 8.61 ± 0.31 ng/ml; *p* < 0.05). However, there was no increase over control in obstructive hydrocephalus (obstructive: 8.65 ± 0.80 ng/ml).

**Conclusions:**

AQP4 has a modulatory effect on ependyma stability and acts in CSF production and reabsorption. Therefore, the increase of AQP4 in the CSF in congenital hydrocephalus could be due to the fact that AQP4 passes from the parenchyma to the CSF and this AQP4 movement may be a consequence of ependyma denudation.

## Introduction

Aquaporin-4 (AQP4) is the principal water channel of the central nervous system [1,2] which is located in the pia mater, the glial limiting border of the cortical surface, the glial end-feet of the subependymal ventricular layer, the basolateral membrane of the ventricular ependyma, the astrocytic end-feet at cerebral blood vessels [1-3]. This specific distribution of AQP4 suggests that it plays a crucial role in water transport at brain–cerebrospinal fluid (CSF) and brain– blood barriers [4,5]. Hydrocephalus is a central nervous system disorder associated with defective CSF turnover, and AQP4 is a water channel located in the areas described above associated with the elimination of cerebral edema [3,6].

Many studies report that there is an up-regulation of AQP4 in animal hydrocephalus models which suggest that this up-regulation may be due to a compensatory effect of hydrocephalus [7-9]. However, this fact has never been verified in human hydrocephalus. Nevertheless, recent studies support the hypothesis that AQP4 participates in the development and integrity of ependyma although the underlying mechanisms have not been clarified [10]. Furthermore, AQP4 is located in the proximity of connexin-43 (Cx43) which is the main component of the gap junctions between ependymal cells [11-14]. Subsequently, authors have reported that AQP4 KO mice presented a significant reduction in the expression of Cx43, a disruption of the gap-junctions in ependymal cells and ependymal disturbance [10]. In addition, AQP4 is functionally linked to Cx43 not only in *in vitro* conditions but also in *in vivo*[10]. Moreover, the abnormal expression of Cx43 could produce ependymal denudation and affect CSF flow [15,16].

Therefore, AQP4 could participate in the physiopathology of the water channels in CSF production, in the adequate formation of the gap-junctions and consequently in the ependyma denudation during ventricular dilation. The aim of the present work is to examine the expression of AQP4 in the CSF in patients with congenital obstructive and communicating hydrocephalus.

## Methods

### Patients and samples

Samples of CSF were taken from the lateral ventricle during first five days of life of thirteen term-pregnancy infants: nine with communicating hydrocephalus and four with obstructive hydrocephalus. Samples of CSF were taken by lumbar puncture at 1, 1, 3, 7 days of age from four control patients with suspected meningitis or encephalitis (Tables 1 and 2). The CSF samples were obtained at the University Hospital of the Canary Islands (La Laguna, Tenerife) and were kept in the Department of Anatomy at the University of La Laguna. CSF samples were collected over the last ten years, centrifuged at 2500 rpm to remove cells and stored at −80°C**.** One of the samples was excluded due to blood contamination. Parents had given verbal informed consent. The medical ethical committee of the University of La Laguna and the University Hospital of the Canary Islands approved the study. The CSF was always extracted for diagnostic and / or therapeutic motives. The diagnosis of the hydrocephalus was determined by ultrasound methods during pregnancy and not controlled by pelvicephalometry just before birth. The type of the hydrocephalus was established by ultrasound methods: tetraventricular hydrocephalus of unknown origin was considered to be communicating hydrocephalus; Dandy-Walker syndrome and Sylvian aqueduct obstruction were considered to be obstructive hydrocephalus (Tables 1, 2).

**Table 1:** Aquaporin-4 in CSF from nine term infants measured by Western-blot

**Patient diagnosis**	**Western-blot analysis (grey scale units%)**	**Age (days)**	**Gender**
COM(A)	41.65	1-5	M
OBS SAO(B)	1.92	1-5	M
COM(C)	34.38	1-5	M
CONT(D)	6.75	1	F
COM(E)	10.37	1-5	M
CONT(F)	7.24	1	M
OBS SAO(G)	21.24	1-5	F
OBS SAO(H)	21.20	1-5	M
COM(I)	73.07	1-5	M

*COM* communicating hydrocephalus, *OBS SAO* obstructive hydrocephalus (Sylvian aqueduct), *CONT* control. Patients A,C,E, and I = communicating hydrocephalus (tetraventricular hydrocephalus of unknown origin); B, G and H = obstructive hydrocephalus (Sylvian aqueduct obstruction); D and F = control.

**Table 2:** Aquaporin-4 in CSF from fifteen term infants measured by enzyme-linked immunoassay (ELISA)

**Patient Diagnosis**	**AQP4 ELISA analysis (ng/ml)**	**Age (days)**	**Gender**
COM(A)	11.41	1-5	M
OBS SAO(B)	7.31	1-5	M
COM(C)	11.24	1-5	M
CONT(D)	8.26	1	F
COM(E)	9.22	1-5	M
CONT(F)	8.66	1	M
OBS SAO(G)	9.06	1-5	F
OBS SAO(H)	10.75	1-5	M
COM(I)	12.15	1-5	M
COM(J)	9.70	1-5	F
COM(K)	14.82	1-5	F
OBS DW(L)	7.47	1-5	M
COM(M)	10.67	1-5	M
CONT(N)	9.46	3	M
CONT(O)	8.06	7	M
COM(P)	9.71	1-5	F

*COM* communicating hydrocephalus, *OBS SAO* obstructive hydrocephalus (Sylvian aqueduct) *OBS DW* obstructive hydrocephalus (Dandy Walker), *CONT* control.

Patients A,C,E,I,J,K,M and P = communicating hydrocephalus (tetraventricular hydrocephalus of unknown origin); B, G, and H = obstructive hydrocephalus (Sylvian aqueduct obstruction); L = obstructive hydrocephalus (Dandy-Walker Syndrome) D,F,N and O = control.

### Western blot analysis

Western blots were performed on the CSF of four communicating hydrocephalus, three obstructive hydrocephalus samples and two control samples. Analysis was performed in triplicate on 35 μl of CSF. Proteins were denatured in a SDS-containing sample buffer (100 mM Tris–HCl pH 6.8, 4% SDS, 2% bromophenol blue, 20% glycerol) and reduced using β-mercaptoethanol. Samples were heated to 95°C for 5 min and run on a SDS-PAGE gel (10% polyacrylamide) for 2 h at 80 V and the proteins were then blotted to a PVDF membrane (Millipore, USA) at 400 mA for 2 h in blotting buffer (39 mM glycine, 48 mM tris-base pH 8.3, 0.037% SDS, 20% methanol). After 1 h blocking (Tris buffer saline-3% BSA), samples were incubated with target antibody at a dilution of 1:1000 for AQP4 (Sigma-Aldrich, USA) in blocking buffer at 4°C overnight. Detection was performed by enhanced chemiluminescence (Amersham Biosciences, UK) after an hour’s incubation with horseradish peroxidase-conjugated anti-rabbit 1:20,000 (Jackson, USA). The primary antibody was omitted to control for the method specificity.

Densitometric analysis was completed in Image J (v. 1.43 u, NIH, Bethesda, MD, USA). The 'Mean Gray Value' was measured on all Western-blot samples. This value gives the average stain intensity in greyscale units for all threshold pixels. The data for triplicate samples were averaged**.** A non-parametric Kruskal-Wallis test was used for data comparison between the control and hydrocephalus groups, which was conducted using the IBM SPSS statistic 19 software where data were considered as statistically significant at *p* < 0.05.

### ELISA assays

In order to give a more accurate quantification of the AQP4 in the CSF of hydrocephalus and control patients, seven additional samples were added to those used for Western blot (Table 2); four communicating hydrocephalus (J,H,M,P), one obstructive hydrocephalus (L) and two control samples (N,O). A commercial enzyme immunoassay (ELISA) was performed (USCN life Science Inc., Wuhan, China) according to the manufacturer’s instructions. Briefly, micro titer plates precoated with AQP4-antibodies were filled with diluted AQP4 standard such as 40 ng/mL, 20 ng/mL, 10 ng/mL, 5 ng/mL, 2.5 ng/mL, 1.25 ng/mL, 0.625 ng/mL. Samples diluted 1:20, were incubated for 2 h at 37°C. After removing the samples and standard, a biotin-conjugated polyclonal antibody preparation specific to AQP4 was added and the plates were incubated for 1 h. After washing cycles, the plates were incubated with avidin conjugated to horseradish peroxidase for 1 h at 37°C. After washing cycles, 3.3’5.5’-tetramethylbenzidine (TMB) was added and incubated at 37°C for 20 min. The enzyme–substrate reaction was terminated with H2SO4. The optical density was measured immediately at 450 nm. According to the manufacturer, no significant cross-reactivity or interference between human AQP4 and other AQP analogues was observed. The non-parametric Kruskal-Wallis test was used for data comparison between the control and hydrocephalus groups. Data were considered as statistically significant at *p* < 0.05 which was conducted using the IBM SPSS statistics 19 software.

## Results

**Figure 1:**
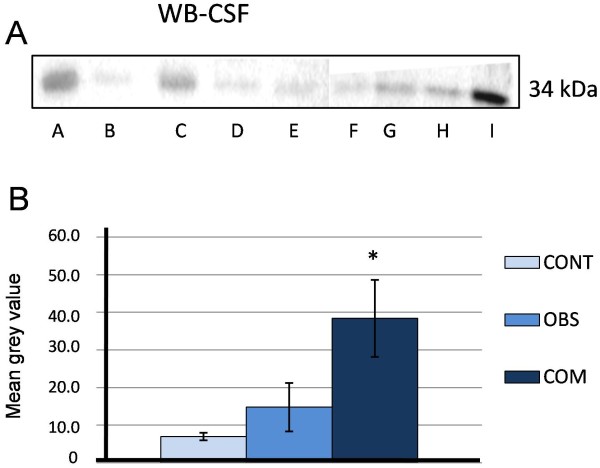
**Western blot for aquaporin-4 for nine CSF samples from control (CONT, n =2), obstructive hydrocephalus (OBS, n = 3) and communicating hydrocephalus (COM, n = 4) in one to five day old term-pregnancy infants; B: Histogram of mean grey value obtained by densitometry of aquaporin-4 by Western blot analysis of the CSF.** * is significantly different from control and obstructive hydrocephalus, *p* < 0.05. Patients A,C,E, and I = communicating hydrocephalus (tetraventricular hydrocephalus of unknown origin); B, G and H = obstructive hydrocephalus (Sylvian aqueduct obstruction); D and F = control.

The Western blots showed only one AQP4 band of 34 KDa in all samples. The mean grey value in CSF was higher in both types of hydrocephalus; obstructive (14.7% mean grey value) and communicating hydrocephalus (38.3% mean grey value) when compared to the control CSF (6.9% mean grey value) (Figure 1A, B, Table 1). The increase over control was significant for communicating hydrocephalus only, p < 0.05.

**Figure 2:**
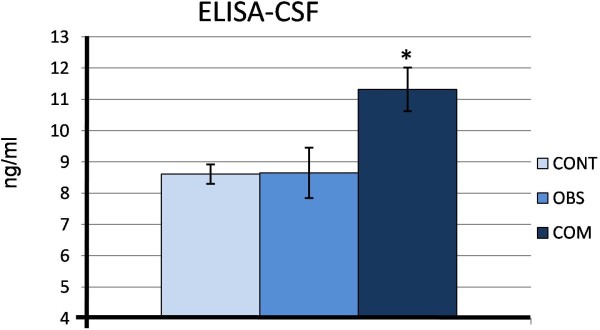
**Histogram of aquaporin-4 in CSF measured by ELISA in control infants (CONT, n = 4), infants with obstructive hydrocephalus (OBS, n = 4) and communicating hydrocephalus (COM, n = 8).** * is significantly different from control and obstructive hydrocephalus, *p* < 0.05.

ELISA also, showed (Figure 2 A and Table 1) that AQP4 was also greater in communicating hydrocephalus when compared to the control (χ ^2^_2_ = 7.14 *p* < 0,05) and when compared to obstructive hydrocephalus (χ ^2^_2_ = 4.32 *p* < 0.05). The average concentration of the controls was 8.61 ± 0.31 ng/ml, of obstructive hydrocephalus was 8.65 ± 0.80 ng/ml and of communicating hydrocephalus was 11.32 ± 0.69 ng/ml.

## Discussion

Aquaporins (AQPs) are membrane proteins that facilitate water and small solute movement in tissues. Hydrocephalus is the main central nervous system disorder associated with defective CSF turnover [3,6]. Aquaporin-4 (AQP4) is a water channel mainly located at the blood–brain barrier (BBB) and is associated with the elimination of cerebral edema via this route [3]. AQP4 levels are significantly altered in kaolin-induced hydrocephalus, suggesting that AQP4 could play an important neurodefensive role in hydrocephalus and CSF disorders [17,18]. Biphasic AQP1 expression in the choroid plexus with increased AQPs 1 and 4 at the brain-fluid interfaces may indicate compensatory mechanisms to regulate choroidal CSF secretion and increase parenchymal fluid absorption in high-pressure hydrocephalus [19].

A study [20] detecting AQP1 and AQP4 in CSF determined that the mean concentration of AQP1 in CSF was significantly higher in patients with bacterial meningitis (BM), AQP4 was also greater but not significantly so [20]. One can see that most of these studies, in hydrocephalus cases, used animal models (AQP4-knockout mice, H-Tx rats, and kaolin and L-α-lysophosphatidylcholine stearoyl-injection models of hydrocephalus), which indicate that there is an up-regulation of AQP4 expression at the BBB interface. Only one study reported sporadic cases of obstructive hydrocephalus in a subgroup of AQP4-knockout mice [3]. But few publications have studied the association between aquaporins and congenital hydrocephalus. Therefore, all existing studies using animal models propose an adaptive and protective role of AQP4 to resolve hydrocephalic edema at the brain barriers in the pathophysiology of hydrocephalus [3].

Thus, despite their inherent relationship in the processes of water transport in the previously mentioned conditions (edema reabsorption, meningitis, hydrocephalus), AQP4, by means of connexin-43 (Cx43) regulation, is also a modulator of the formation and development of the gap junctions in the ependymal basolateral membrane and astrocyte feet. It has been reported that knockdown and knockout AQP4 mice downregulate the expression of Cx43 in different *in vitro* studies [10,21,22]. Furthermore, AQP4 not only regulates the expression of Cx43, but also secondarily intervenes in the ependyma denudation, given that the decrease of gap junctions, in which Cx43 is the main protein [15,16].

The CSF protein concentration is compartmental related, thus in the lumbar compartment the protein concentration is higher than in the ventricular compartment [23]. Despite that, in results presented here the AQP4 was increased in the CSF of the hydrocephalus samples taken from the lateral ventricle compared to control CSF taken from lumbar puncture. This increase in CSF AQP4 in hydrocephalus may occur as a consequence of the loss of communication between ependymal cells and subsequent denudation of ependyma, when AQP4 would pass into the CSF. This ependymal loss is accompanied by a microglial and astroglial cell reaction; the subependymal astroglial cells respond by proliferation in such a way that they form a glial scar-like covering of the ventricular surface to replace the ependyma [24,25]. At that time, if cellular death or disruption occurs, the AQP4 could be in contact with the ventricular lumen and may pass into the CSF. Furthermore, the occurrence of various stages of ependymal denudation within full-term spina bifida aperta (SBA) fetuses, suggests that there may be continuation of the process after birth and that cases of communicating hydrocephalus could soon develop into non-communicating hydrocephalus [16]. Therefore, the increase of CSF AQP4 may be a consequence of the ependymal denudation and the level of AQP4 in the CSF could be an indicator of the ependyma status and the hydrocephalus stage.

## Conclusion

We have shown that the AQP4 concentration is higher in the CSF of communicating hydrocephalus infants than in the CSF of non-communicating hydrocephalus patients or controls. It is possible that AQP4 may freely leak from the parenchyma to the CSF during the early stages, when ependymal denudation is occurring and the hydrocephalus remains communicating. The leaking of AQP4 to the CSF may decrease as a glial scar-like layer covers the ventricular surface and subsequent stenosis and obstruction occurs.

## Competing interests

There are no conflicts of interest.

## Authors’ contributions

LCR and IGM participated in conception and design of the study and in the collection of data. JMGT, ACR, HPC participated in performing the methods and contributed to analysis and interpretation of data. LCR, IGM, ACP and ECC participated in conception, design manuscript, analysis and interpretation of data and preparation of the final version. All authors have read and approved the final version of the manuscript.
